# 1Controlled evaluation of a neurofeedback training of slow cortical potentials in children with Attention Deficit/Hyperactivity Disorder (ADHD)

**DOI:** 10.1186/1744-9081-3-35

**Published:** 2007-07-26

**Authors:** Renate Drechsler, Marc Straub, Mirko Doehnert, Hartmut Heinrich, Hans-Christoph Steinhausen, Daniel Brandeis

**Affiliations:** 1 Department of Child and Adolescent Psychiatry, University of Zurich, Switzerland; 2 Child & Adolescent Psychiatry, University of Erlangen-Nürnberg, Germany; 3 Heckscher Klinik, München, Germany; 4 Center of Integrative Human Physiology University of Zurich, Switzerland

## Abstract

**Background:**

Although several promising studies on neurofeedback training in Attention Deficit/Hyperactivity Disorder (ADHD) have been performed in recent years, the specificity of positive treatment effects continues to be challenged.

**Methods:**

To evaluate the specificity of a neurofeedback training of slow cortical potentials, a twofold strategy was pursued: First, the efficacy of neurofeedback training was compared to a group training program for children with ADHD. Secondly, the extent of improvements observed in the neurofeedback group in relation to successful regulation of cortical activation was examined. Parents and teachers rated children's behaviour and executive functions before and after treatment. In addition, children underwent neuropsychological testing before and after training.

**Results:**

According to parents' and teachers' ratings, children of the neurofeedback training group improved more than children who had participated in a group therapy program, particularly in attention and cognition related domains. On neuropsychological measures children of both groups showed similar improvements. However, only about half of the neurofeedback group learned to regulate cortical activation during a transfer condition without direct feedback. Behavioural improvements of this subgroup were moderately related to neurofeedback training performance, whereas effective parental support accounted better for some advantages of neurofeedback training compared to group therapy according to parents' and teachers' ratings.

**Conclusion:**

There is a specific training effect of neurofeedback of slow cortical potentials due to enhanced cortical control. However, non-specific factors, such as parental support, may also contribute to the positive behavioural effects induced by the neurofeedback training.

## Background

Although stimulant medication has proven as the most efficacious strategy in the treatment of ADHD, there is a considerable need for effective treatment alternatives to help the sizeable number of children who do not respond to medication, suffer from intolerable side effects or whose parents are reluctant to administer stimulant medication to their children. In addition, stimulant medication seems to alleviate primary symptoms of ADHD in children, but long-lasting effects on the underlying pathology are uncertain. Neurofeedback is arguably the most promising alternative treatment for patients with ADHD [1]. Clinical trials have been described since the seventies [2], but controlled studies on the effectiveness of neurofeedback training in children with ADHD have only been published in recent years (see [1,3,4], for reviews). Positive effects of neurofeedback training have been described on primary symptoms of ADHD as rated by parents, on cognitive testing, and, in some studies, on teachers' ratings of behavioural symptoms [5-10].

The vast majority of studies on neurofeedback in ADHD have examined the training of EEG (electroencephalogram) frequency bands. In frequency training, the participants learn to increase or decrease a predetermined ratio of EEG frequencies at a certain electrode position. The most often employed training rationale with ADHD patients is to decrease the activity of the theta band and to increase the activity of the beta band, thus aiming at a more attentive state. As long as the participant succeeds in regulating his cortical activity within the desired range, a signal – auditory, visual or both – is fed back. A second training form is the training of slow cortical potentials (SCPs). SCPs are event-related changes of cortical activity lasting from several hundreds milliseconds to several seconds [11]. Participants learn to increase positivity or negativity over their sensorimotor cortex, typically as measured by a central midline electrode (Cz). Slow central negativity is also found during cognitive preparation and has been related to increased cortical activation of a network including the central region underneath the feedback electrode, while central positivity may reflect a corresponding decrease or inhibition of activation [11,12]. On trials where the change in the desired direction reaches a critical threshold, a positive reinforcement signal is fed back to the participant. This type of training is aiming more directly at the control of cortical regulation and at the efficient allocation of resources, which is supposed to be impaired in ADHD [13,14]. Training of SCPs has been successfully employed in the treatment of epilepsy (e.g. [15,16]). Recently, studies on SCP neurofeedback training with children with ADHD have been published with encouraging results [7,8,10].

Although there is growing evidence for the efficacy of neurofeedback training, there is still some scepticism due to the methodological issues in studies published so far (e.g. see [17]). Two major methodological challenges are to prove convincingly that specific aspects of neurofeedback therapy mediate positive effects and that these effects are comparable or superior to appropriate control treatments. According to certain critics it is questionable whether positive effects after neurofeedback training can be related to the improved cortical regulation or rather to unspecific treatment effects. As principles of behavioural therapy, such as systematic reinforcement and a positive relationship to a therapist, are integrated parts of the neurofeedback training especially with children, selective effects of the EEG feedback are difficult to isolate. It has also been objected that other feedback mechanisms than electrophysiological ones, such as respiratory or muscular feedback, can be involuntarily involved and may contribute to improved behavioural control or serve as a relaxation training [18]. As the transfer of acquired strategies from the training setting into daily life may also depend on a favourable environment, the parenting style might represent a significant factor for improvement or interact with more specific effects (see [6]).

Major arguments in favour of the specificity of training effects come from studies that relate behavioural improvement directly to electrophysiological parameters of the training performance. In their SCP study, Strehl et al. [10] divided participants into groups of successful or unsuccessful regulators, according to the mean EEG-amplitude achieved during negativity trials, with large amplitudes indicating successful regulation. Children who had successfully learned to regulate cortical activity showed a better clinical improvement at the end of training than the unsuccessful regulators. Another argument in favour of specific treatment effects is provided by studies on relevant changes observed in EEG or ERP (event-related potential) data of the participants with ADHD before and after neurofeedback training. Kropotov et al. [19] report improved ERPs in a go/nogo paradigm after a frequency neurofeedback training in a group of "good performers". Normalization of spontaneous EEG after frequency neurofeedback training was found by Monastra et al. [4] but not by Carmody et al. [20]. After a SCP training, Heinrich et al. [7] reported a selective increase of the contingent negative variation in the ERP during the cued Continuous Performance Test (CPT), suggesting improved preparation and increased allocation of attention.

Another critical point is the choice of control groups. Without an appropriate control it is difficult to conclude whether significant behavioural change or improvement in neuropsychological tests can be related to the administered treatment, or to unspecific factors such as maturation, changes in the parental attitude, to enhanced awareness of problem behaviour or to simple retest effects. However, for ethical or methodological reasons, proper controls are difficult to find. Several studies have used waiting groups (e.g. [20,21]) which from an ethical point of view is not an optimal solution and may present a bias because of maturational changes, multiple testing or changed parental attitudes. Other studies compared neurofeedback with stimulant therapy (e.g[9,22,23]). Heywood & Beale [18] used intermittent placebo feedback as a control condition, where signals are fed back at random. Although the participants had been previously informed that this might occur during the training, the procedure seems problematic especially with children.

The aim of the present study was to evaluate the efficacy of a SCP neurofeedback training in children with ADHD. In order to control for unspecific effects, we opted for a double strategy: First we hypothesised that participants of a neurofeedback training should improve their ability to regulate their cortical activation over time, as represented by an increase of the mean amplitude in positive direction during positivity trials and in negative direction during negativity trials. As an improved attentional mental set has been related to enhanced negativity, changes in the regulation during negativity trials were considered more relevant for training success in ADHD. It was further assumed that behavioural changes as rated by parents and teachers and improvement on cognitive tests before and after treatment should be related to the learned ability to regulate cortical activation. As a second strategy, a different treatment was introduced, controlling for retest effects during neuropsychological testing and for unspecific changes which could also be induced by other types of behavioural training. It consisted in a group therapy program for children with ADHD which was comparable to the neurofeedback training in intensity and duration. Although behavioural therapy or behaviour management have proven beneficial for the treatment of relevant problem areas in children with ADHD, such as social interaction, self-management or self-esteem ([24-26]; see also [27]) their immediate effects on basic symptoms of ADHD are questionable, e.g. [28]. Neurofeedback, in contrast, is supposed to aim more directly at the underlying neurobiological dysfunction. Therefore we expected a stronger effect of neurofeedback training on ADHD core symptoms rated on clinical scales and on cognitive performance than from a more indirect form of treatment.

## Methods

### Participants

Seventeen children with ADHD, 13 boys and four girls, participated in a neurofeedback-training program. The control group consisted of 13 children with ADHD, 10 boys and three girls, who participated in a group training program (see Table 1 for a description of the sample). Group differences were statistically not significant. Randomization of group assignment was incomplete, as certain therapeutic and practical aspects had to be respected, e.g. the age range within the children participating in the group program had to be small, in a mixed group at least two girls had to participate, and children and parents of the neurofeedback group had to be available during vacation for intense trainings. Some children, respectively their parents, showed clear preferences for one type of training or wished to participate at both types of trainings. In these latter cases only data from the first treatment were entered into the analysis.

**Table 1:** Description of the groups

	Neurofeedback	Group therapy	
	N = 17	N = 13	p
Boys/girls	13/4	10/3	n.s.
Age			
mean (SD)	10.5 (1.3)	11.2 (1.0)	n.s.
range	9.1–12.8	10.1–13.1	
IQ			
mean (SD)	101 (10.3)	110 (19.2)	n.s.
Diagnosis (DSM IV)			n.s.
Combined	11	8	
Inattentive	5	5	
Hyperactive-imp.	1		
Stimulant medication	6	6	n.s.
CBCL (t-scores)			
Internalizing	60.5 (10.7)	55.0 (11.3)	n.s.
Externalizing	61.2 (10.7)	58.8 (9.9)	n.s.

Children were recruited in the Department of Child and Adolescent Psychiatry, University of Zurich, or through advertisements placed on the homepage of the clinic. Children had to fulfil the following criteria:

- Age between 9 to 13 years

- Formal diagnosis of ADHD before entering the study

- IQ > 80

- No known neurological disease

Children currently taking stimulant medication were not excluded from the study, but their parents were asked to keep treatment conditions constant throughout the training period in order to avoid interfering medication effects. All the children stopped medication at last 24 h. prior to neuropsychological testing. Informed consent was obtained by all the parents and children. Clinical diagnosis and subtyping was confirmed by HYPESCHEME, a computerized operational criteria checklist and diagnostic algorithm for DSM-IV and ICD-10 which includes a diagnostic interview (PACS: [29]; see [30]). When discrepancies occurred between HYPESCHEME and previous classification, which was the case for two children, diagnosis was based on judgements by two independent experienced clinicians.

### Materials

#### Behavioural ratings

Parents rated children before and after the training on a German standardized DSM IV- questionnaire for ADHD (FBB-HKS) [31], on the Conners' Parent Rating Scale (CPRS) [32] and on the Behavior Rating Inventory for Executive Function (BRIEF) [34]. To screen for comorbid clinical conditions, the Child Behavior Checklist (CBCL) [35] was completed only once by parents, before the training. At the end of the training, parents participated in a semi-standardized interview, conducted by a psychologist who had not been involved in the therapy. Teachers rated children before and after the training on the Conners' Teacher Rating Scale (CTRS) [33] and on the teacher's version of the BRIEF [34].

#### Neuropsychological evaluation

A short form of the German WISC III [36] was used to estimate IQ, with Block Design, Vocabulary, Picture Arrangement and Arithmetics. Before and after the training, all children were tested using a comprehensive battery of neuropsychological tests:

- "Alertness", a subtest from the computerized standardized test battery "Test for Attentional Performance" (TAP 1.7) [37]. In this task, participants respond to a visual stimulus by pressing a response button as fast as possible. A condition with auditory warning signal, where subjects are supposed to react faster, is contrasted with a condition without warning tone. Standard deviations of response time from both conditions are taken in this study as measures of response time variability.

- "Go/nogo", a subtest from TAP [37]. In this inhibitory control task, subjects respond as fast as possible to a go-stimulus, an "X" presented on the computer screen, by pressing a response button, and inhibit responding when the no/go-stimulus, a "+", appears.

- "D2" [38]. In this paper-pencil – test of focussed and selective attention subjects cross out critical letters on a working sheet, working line by line. They dispose of 20 seconds for finishing each line. The concentration performance is calculated as the number of correctly cancelled minus the total number of incorrectly cancelled items.

- "Score!", a subtest from the Test of Everyday Attention for children (Tea-ch) tapping sustained attention [39]. In this task children have to count the number of auditory signals which they hear on a CD and which are presented at irregular intervals.

- "Code transmission" (Tea-ch). In this sustained attention task, children listen to a series of numbers from a CD. Whenever the critical number "five" appears twice, subjects have to name the number that immediately came before the fives. Test duration is twelve minutes.

- Trail Making Test (TMT) for children. This paper pencil task is a simpler version of the well-known TMT procedure [40]. In part A, numbers spread randomly on a sheet have to be connected in ascending order as quickly as possible. In part B, numbers and letters of the alphabet have to be connected in ascending order, switching continuously between letter and number. Time differences between part A and part B are considered as indicative of switching costs.

In addition children participated pre- and post-training in an EEG and ERP recording. These results will be reported elsewhere [41].

### Treatments

#### SCP neurofeedback training

Children were trained with the neurofeedback system "Goefi" which had been developed for the training of children with ADHD (see [7]). The training was presented like a computer game for children with well-known cartoon figures from a TV show as protagonists. The child was instructed by different colours to activate or deactivate cortical excitability, the colour "blue" indicating decrease (positivity) and the colour "red" indicating increase of cortical activation (negativity). Successful activation or deactivation was fed back by an action of the animated cartoon figure (i.e. a pole vaulting mouse) and rewarded by points.

A feedback-trial consisted of a baseline period of 2 seconds, where the child was instructed to activate (negativity trials) or to deactivate (positivity trials), and a feedback period of 6 seconds, which started with an acoustic signal. During the feedback period, the feedback signal was updated every 100 ms. The reinforcement described above was provided at the end of the feedback period. The interval between trials was fixed to 5 ± 1 seconds. Negativity (50%) and positivity trials (50%) were presented randomly. Feedback was measured from Cz, with reference on mastoids, a bandwidth from .01–30 Hz, and a sampling rate of 250 Hz. Artefacts due to vertical eye movements were measured by two electrodes above and below the left eye and were corrected online. Increase or decrease of activation exceeding a threshold previously defined (200 μV for the eye channel, 100 μV for the EEG-channel; see [7]), were considered as artefacts and were not fed back to participants.

In addition to feedback-trials, children also performed transfer trials with instructions given on the computer screen and with EEG recording, but no feedback was provided on the screen (= EEG transfer training). The aim of the EEG transfer trials was to prepare for generalization of learned activation or deactivation into daily-life situations (see [8]). In a second form of transfer training children trained with red and blue transfer cards (= transfer training with cards) and without EEG recording. They were encouraged to carry these cards with them and to use them in everyday settings, e.g. during homework or during school exams.

Each training course consisted of 30 sessions. Two training sessions of 45 minutes each were administered on the same day, usually as double sessions, according to the following protocol: Children started with a block of 40 feedback-trials, in randomized order for positivity and negativity, followed by a block of 30 EEG transfer trials and by a block of 40 transfer trials with training cards. After a break of 15 to 20 minutes another block of 40 feedback-trials started, followed by a block of 30 EEG transfer-trials. The training was conceived according to cognitive behavioural principles, with therapeutic interaction between trainer and child being fundamental. The trainer encouraged the child to develop an appropriate strategy in order to induce activation or deactivation, to work out a plan how and where to use the strategy in daily life, discussed the problems encountered with transfer and introduced a training diary. Information of the parents represented another important part of the training. Parents were invited to participate at training sessions and to supervise transfer training with cards at home.

The training started as a two weeks-vacation-course with daily training sessions. This intense training phase was followed by a break of 5 weeks. Children and parents had been instructed previously to train regularly during this time with transfer cards and to employ the learned strategies in everyday-life situations. During these weeks, the trainer held contact with children and families by phone and e-mails. The second feedback-training period consisted of 5 double sessions, conducted once or twice a week during the school semester over a time of three weeks. Parents' involvement (low vs. high) based upon the contact held during the transfer period was rated by the trainer. Pilot studies had indicated that parents had to assume a very active role in the neurofeedback program, e.g. to remind the child during the transfer period without EEG training to exercise with transfer cards or to encourage the use of transfer cards in everyday situations. Parents were explicitly instructed to reward training efforts with "Goefi"-points which could be exchanged against small presents. Effective parental support (high vs. low) was thus assessed in a semistructured interview by asking parents whether they had reminded the child to exercise, had actively supported the use of transfer cards and had used the reward – system.

#### Group therapy

**Figure 1:**
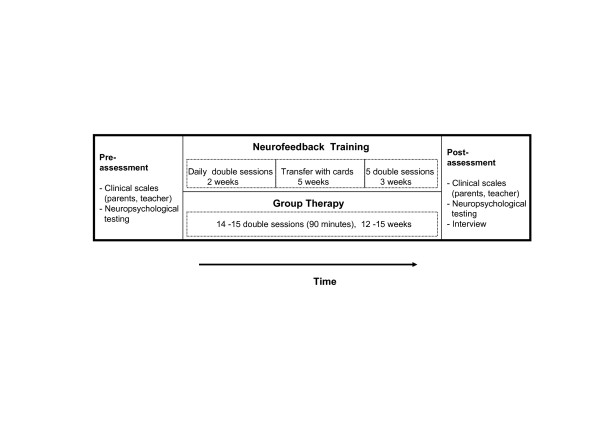
Design of the study

The group training program for children with ADHD was based on principles of cognitive behavioural therapy and conducted by two experienced child psychologists. It comprised components of social skill training, self-management, metacognitive skill training and enhancement of self-awareness. A major focus was on how to give and to benefit from feedback. Some modules were taken from or inspired by published programs (e.g. [42,43]), others had been newly developed. A group of five to six children participated in each group therapy program which consisted of 14 to 15 sessions of ninety minutes, hold with a frequency of one or two sessions per week. A summary of the training design of both treatments is shown in Figure 1.

Parents were invited to participate at the last 15 minutes of each session and received information on program issues on additional parent meetings. Parental involvement (high vs. low) was assessed based on the frequency of participations at treatments. In order to stabilize learning effects of the group sessions, children were assigned with "homework" from one week to the next, such as to measure the duration of all kinds of everyday activities with a stop watch in order to improve time estimation. Parents received information on the objective of these tasks without being explicitly asked to supervise them. In group therapy positive behaviour was also rewarded by points, but only during therapy sessions. Therefore the importance of the commitment shown by the parents in both groups was not directly comparable, and effective parental support at home could not be assessed.

Although therapeutic setting, method, and the distribution of sessions over time are different, the two treatments present a number of similarities: Both treatments are behavioural therapies aiming at the enhancement of self-awareness and self-control. Both types of treatments were roughly comparable in duration and in the amount of time spent in therapy sessions. In both treatments parents regularly received information on ongoing treatment issues and were invited to collaborate.

### Statistical analysis

Repeated measures MANOVAS were conducted in order to analyse how behavioural scales and neuropsychological test performance changed with treatment. Relations between training performance (defined as the mean differentiation between positivity and negativity achieved during transfer trials) and outcome and between parental support and outcome were analysed by correlations.

## Results

### Effects of neurofeedback training compared to group therapy

#### Behavioural ratings

Composite scores of three behavioural scales rated by parents were entered into a repeated measures MANOVA (group by time by six composite scores – FBB-HKS: Hyperactivity, Impulsivity, Inattention; CPRS: Global Score; BRIEF: Behavioural Index, Metacognition Index). A significant effect for time (p = .011, partial Eta squared = .484) and an interaction of group by time (p = .004, partial Eta squared = .507) but no significant group differences were found (Table 2). The group by time interaction remained significant for the Inattention score of the DSM IV Rating Scale (FBB-HKS) and the Metacognition Index of the BRIEF after Bonferroni correction (Table 2). Post hoc t-tests revealed that children of the neurofeedback group improved significantly on the Inattention score (t = 3.957, p = .001) and on the Metacognition Index (t = 3.368, p = .004) whereas no changes were observed in the group therapy children on either subscale (Inattention score t = -.557, p = .588, Metacognition Index t = -1.035, p = .321). Therefore, according to parents' ratings, children benefited more from neurofeedback training than from group therapy on two subscales related to attention and cognitive performance. Similar advantages for neurofeedback over group training were found on the Impulsivity scale and for the Global Index of the CPRS, but these did not survive the conservative Bonferroni correction. Among the significant changes before correction, only those on the Hyperactivity subscale did not differentiate between the two groups. In addition, when effects sizes of changes on the CPRS Global Score were calculated separately for both groups, it could be shown that changes within the neurofeedback group were responsible for the improvement on the CPRS Gobal Index (neurofeedback: partial Eta squared = .557; group therapy: partial Eta squared = .155). One might thus question whether any behavioural improvement after group therapy can be found at all.

**Table 2:** Behavioural scores before and after neurofeedback training or group therapy

	**Neurofeedback**		**Group therapy**								
	(N = 17)		(N = 13)								
	Time 1	Time 2	Time 1	Time 2	Group		Time		Group by Time		
					F,	p	F,	p	F,	p	
**Parent Ratings**											
**FBB-HKS (DSM IV)**											
Hyperactivity	1.21 (60)	0.75 (46)	.98 (58)	0.80 (.67)	.190	.666	6.974	.013	1.395	.247	
Inattention	2.07 (.45)	1.41 (.49)	1.50 (.65)	1.57 (.67)	1.354	.254	6.535	.016	10.542	.004 °	NF>GT
Impulsivity	1.19 (.82)	0.85 (.56)	1.01 (.58)	1.24 (.94)	.183	.672	.190	.666	4.783	.037	
**CPRS**											
Global index	16.1(4.9)	10.7 (4.5)	12.0 (5.2)	10.3 (5.1)	1.976	.171	17.382	.000 °	4.765>	.038	
**BRIEF parents**											
Behavioural Index	51.3 (10.4)	47.0 (10.2)	47.8(13.2)	44.5 (11.2)	1.212	.280	4.157	.052	.122	.730	
Metacognition Index	99.5 (15.0)	88.6 (13.6)	84.2 (19.4)	86.5 (20.8)	.379	.543	4.491	.043	9.973	.004 °	NF>GT
											
**Teacher Ratings**											
**CTRS**											
Global Index	10.9(6.2)	9.6(5.7)	10.7 (6.7)	11.4 (5.9)	.148	.703	.117	.734	1.553	.223	
**BRIEF teacher**											
Behavioural Index	46.8 (10.4)	44.1 (10.2)	32.7(13.2)	29.4 (11.2)	7.612	.010 °	1.423	.243	.0	.999	
Metacognition Index	93.3 (15)	78.3 (13.6)	85.7(19.4)	87.5(20.8)	.062	.813	4.268	.048	6.799	.014	NF>GT

FBB-HKS (= DSM IV Checklist), severity scores, CPRS Conners' Parent Rating Scale, CTRS Conners' Teacher Rating Scale, BRIEF Behavior Rating Inventory of Executive Function. NF = Neurofeedback, GT = Group Therapy.

MANOVA parent ratings (group by time by 6 subscales): group F = .512, p = .793; time F = 3.603, p = .011; group × time F = 3.935, p = .007; df hypothesis = 6, df error = 23.

MANOVA teacher ratings (group by time by 3 subscales): group F = 3.292, p = .036; time F = 1.595 p = .215; time by group F = 5.318 p = .005; df hypothesis = 3, df error = 26. ° = p <.05 when corrected with Bonferroni

Composite scores of the behavioural scales CTRS and BRIEF rated by teachers before and after training were entered into another repeated measures MANOVA (group by time by three scores). The group effect and the interaction of group by time were found to be significant (group p = .036, partial Eta squared = .275; group by time p = .036, partial Eta squared = .380) (Table 2), while no general improvement over time was observed. In post hoc analyses the group effect was significant for the Behavioral Index of the BRIEF. On this index teachers had rated children of the neurofeedback group as more impaired than the children of the therapy group at both assessments (before training t = 2.344, p = .004, after training t = 2.924, p = .007). A significant group by time interaction was observed for the Metacognition Index of the BRIEF: Teachers rated changes related to cognitive performance as more pronounced after neurofeedback than after group therapy.

#### Neuropsychological performance

Neuropsychological test performances were entered into a repeated measures MANOVA (group by time by 7 performance parameters) (Table 3). Both groups showed a general improvement over time (time p = .000, partial Eta squared = .751), which was significant in post hoc tests (ANOVAS) for the number of commission errors in the Go/nogo test, the concentration performance in the cancellation task (D2), and the switching speed in the Trail Making Test. Interaction of time by group was not significant, indicating that with regard to neuropsychological performance both groups improved similarly over time. Group differences were significant (p = .013, partial Eta squared = .520), but there was only one significant performance difference between groups, the number of commission errors in the Go/nogo task, where children of the neurofeedback group made significantly more errors. These group differences were highly significant before training (t = 3.182, p = .004) and showed a strong trend (t = 2.012, p = .054) after the training. Effect sizes of significant changes over time were calculated separately for both groups. Effects were moderate to large in the neurofeedback group (TAP Go/nogo: partial Eta squared = .616; D2 concentration performance: partial Eta squared = .555; TMT: partial Eta squared = .339) and small to moderate after group therapy (TAP Go/nogo: partial Eta squared = .389; D2 concentration performance: partial Eta squared = .301; TMT: partial Eta squared = .245).

**Table 3:** Results of neuropsychological tests before and after neurofeedback training and group therapy

	**Neurofeedback **(N = 17)		**Group therapy **(N = 13)							
	Time 1	Time 2	Time 1	Time 2	Group		Time		Group by Time	
	Mean (SD)	Mean (SD)	Mean (SD)	Mean (SD)	F,	p	F,	p	F,	p
**TAP, Alertness**	71 (30)	74 (41)	80 (36)	72 (60)	.057	.813	.057	.814	.313	.580
SD RT without warning (msecs)	68 (39)	64 (30)	71 (48)	81 (72)	.394	.536	.133	.719	.925	.344
SD RT with warning (msecs)										
**TAP, Go/nogo**										
Errors (commission)	12.1 (4.8)	4.6 (3.0)	6.5 (5.3)	2.3 (3.3)	13.634	.001 °	29.691	.000	3.060	.099
**Score **(Tea-ch)										
Correct responses	22.3 (19.8)	41.4 (26.7)	30.0 (28.6)	38.6 (34.8)	.502	.444	3.861	.059	3.099	.094
**Code transmission **(Tea-ch)										
Correct responses	35.3 (2.8)	36.4 (3.1)	36.0 (4.0)	36.0 (3.5)	.041	.842	1.015	.322	1.015	.322
**D2**										
Concentration performance	92 (25)	125 (48)	119 (62)	152 (31)	3.479	.073	19.210	.000	.014	.908
**Trail Making Test**										
Time B -Time A (secs)	25.0 (17.4)	12.3 (5.1)	27.0 (15.2)	18.7 (13.4)	1.240	.275	11.224	.002 °	.486	.491

TAP = Test for Attentional Performance, Tea-ch = Test of Everyday Attention for children, D2 = D2 Test of Attention, SD RT = Standard deviation of reaction time, ° = p <.05 when corrected with Bonferroni.

MANOVA group by time by test results: group F = 3.404, p = .013, time F = 9.473, p = .000; group by time F = 1.162, p = .364; hypotheses df = 7, error df = 22.

### Effects of the neurofeedback training on cortical regulation and behavior

#### Cortical self-regulation after SCP neurofeedback training

**Figure 2:**
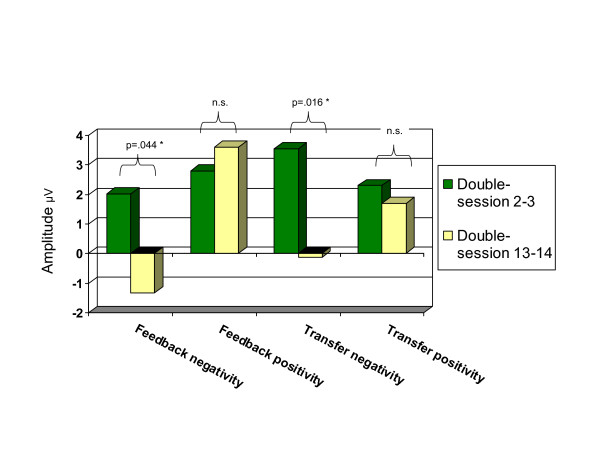
**Changes of mean amplitudes between beginning and end of the training**. Differences of the mean amplitudes between the beginning (double session   2–3) and the end (double-session 13–14) of the training during   negativity or positivity trials in feedback and transfer conditions are   represented in Figure 2. (N = 17)

The cortical activation during the sessions at the beginning of the training (double sessions 2 and 3) was compared to sessions at the end of the training (double sessions 13 and 14) in order to evaluate changes in cortical regulation. To this end the mean amplitudes of positivity and negativity trials were calculated separately for feedback and transfer and entered into a repeated measures MANOVA (time by 4 parameters). In order to reduce effects of fatigue and diminishing motivation towards the end of the session, only the first two trials of each category per double session were considered. For two children who showed a high number of artefacts during the first sessions, data from invalid initial transfer trials were replaced using subsequent valid trials at day 2 and day 3. In another case transfer data of positivity trials could not been reliably obtained at time 1 because of problems with artefacts, and the group mean was entered into the analysis instead. The main effect of time was significant (F = 5.035, p = .011). Post hoc test showed that the mean amplitudes during negativity trials, during feedback (F = 4.772, p = .044) as well as during transfer (F = 7.222, p = .016), showed a significant change in the desired direction (Figure 2). Therefore, it can be concluded that in the course of the training children learned to increase slow central negativity. In contrast, no significant changes of the mean amplitudes during positivity trials were observed (feedback F = .193, p = .666; transfer F = .133, p = .720). Figure 2 illustrates that from the beginning of the training a majority of children spontaneously induced positive amplitudes during both types of trials. Obviously children did not need to learn to further increase positive amplitudes throughout the training but rather to differentiate successfully between negativity and positivity trials. To test this hypothesis, the differentiations between negativity and positivity trials at day 2/3 and 13/14 were compared in an additional analysis: A significant increase was found for the feedback condition (mean difference between amplitudes of positivity and negativity trials, time 1 = .41 μV, time 2 = 4.7 μV, t = -2.59, p = .020), whereas the differentiation in transfer conditions did not significantly increase over time (mean difference time 1 = 1.12 μV, time 2 = 3.149 μV, t = 1.440, p = .169).

#### Individual differences in learning to regulate cortical activation

**Figure 3:**
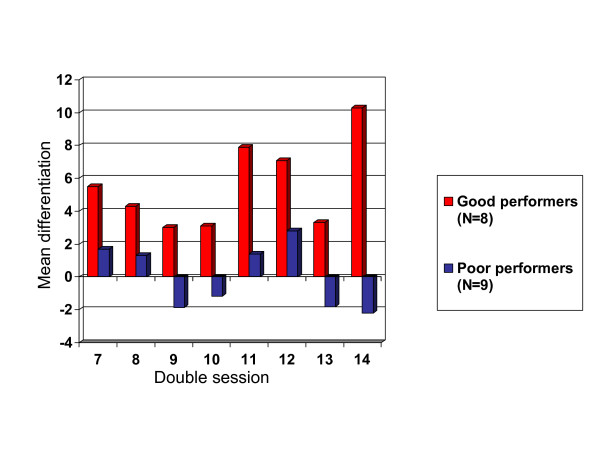
Course of mean differentiation between positivity and negativity trials in double-sessions 7 to 14 of good and poor performers.

In additional analyses the ability to differentiate between states of cortical activation in the transfer condition (which is more demanding than under direct feedback) was used as the relevant index of training success. This ability was considered more representative of improved cortical control in real life situations than a performance that is confined to a laboratory situation. To counterbalance for individual variations from one session to the next, which were considerable throughout the training, and for problems with artefacts at the beginning, the transfer trial data from double sessions 7 to 14 were entered into the analysis. Over all these sessions the group produced a mean difference between amplitudes of positivity and negativity trials of 2.711 μV (SD= 3.630). On closer inspection of the data, it became evident that only some of the children had learned well to differentiate between positivity and negativity in transfer condition. When the children were divided into two subgroups according to the median split of difference amplitudes, a group of good performers (N = 8) with a mean differentiation of 5.72 μV (SD 2.5) could be distinguished from a subgroup of poor performers (N = 9) with a mean differentiation of .034 μV (SD 1.9). The group of poor performers did not learn to differentiate between different states of cortical activation over the training and occasionally continued to produce the opposite of the desired state, which is indicated in Figure 3 by negative mean differences.

To summarize, although an overall group effect was found for an increase in the regulation of negativity during feedback and transfer throughout the training phase, the training did not result in an overall improvement in the differentiation between negative and positive activation in the full group. Only a subgroup of good performers improved. When demographic data of good and poor performers were compared, no salient difference emerged (see Table 4), though, as a tendency, a higher proportion of girls and of children with ADD was present among the poor performers. Otherwise, both subgroups seemed more or less comparable in regard to IQ, parental support, medication, age or initial severity of ADHD – symptoms.

**Table 4:** Comparison of good and poor performers (differentiation in EEG transfer condition)

	**Good performers **(N = 8)	**Poor performers **(N = 9)	p (Mann Whitney U)
Girls/boys	1 (13%)/7 (87%)	3 (33%)/6 (66%)	n.s.
Stimulant	2 (25%)	4 (44%)	n.s.
ADHS/ADS/HS	5 (62%)/2 (25%)/1(12.5%)	5 (55%)/4 (44%)	n.s.
IQ (mean)	100.4 (SD 9.7)	100.9 (SD 9.2)	n.s.
Age (mean)	10.7	10.3	n.s.
High parental support	4 (50%)	6 (66%)	n.s
CBCL Internalizing	60.2 (11.5)	60.8 (9.5)	n.s.
CBCL Externalizing	60.4 (14.4)	61.9 (6.6)	n.s.
Initial ADHD			
symptoms (FBB-HKS)			
Hyperactivity	1.24 (SD .70)	1.18 (SD .54)	n.s.
Inattention	2.09 (SD .57)	2.05 (SD .34)	n.s.
Impulsivity	1.06 (SD 1.15)	1.31 (SD .38)	n.s.
ADHD symptoms after training (FBB-HKS)			
Hyperactivity	.79 (SD .49)	.67 (SD .46)	n.s.
Inattention	1.41 (SD. 54)	1.41 (SD .47)	n.s.
Impulsivity	.82 (SD .59)	.87 (SD .58)	n.s.

ADHS combined subtype, ADS inattentive subtype, HS hyperactive-impulsive subtype

n.s. non significant

#### The relation of behavioural and neuropsychological improvement to training performance

**Figure 4:**
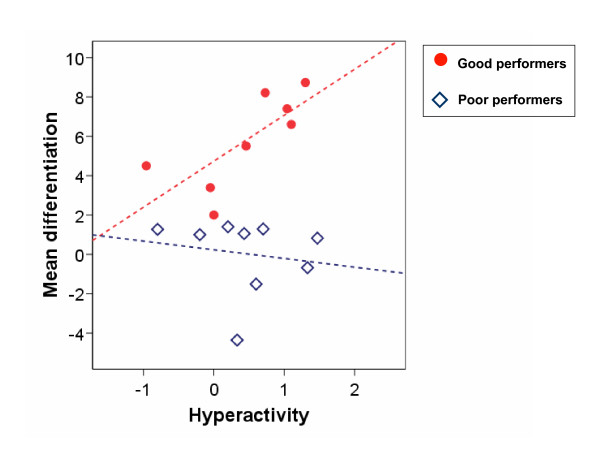
**Mean differentiation in relation to changes of the Hyperactivity score**. The scatterplot represents the mean differentiation between negativity and positivity trials (transfer condition, double-sessions 7 to 14) in relation to changes on the Hyperactivity subscale (FBB-HKS) (time 1 minus time 2) in good (N = 8) and poor performers (N = 9).

To evaluate the relation between training performance and clinical improvement, training parameters were correlated with improvement on behavioural scales and neuropsychological tests. Improvement was conceptualized as difference between time 1 and time 2 (score 1 minus score 2) and correlated with the mean difference of negativity and positivity trials amplitudes in EEG transfer condition of double session 7 to 14 (Table 5). None of these correlation provided significant results, indicating that behavioural improvement cannot simply be related to training performance in EEG transfer condition. However, when correlations were computed for the subgroups of good and poor performers separately, significant correlations emerged for the good performers between the ability to differentiate between negativity and positivity and improvements on the Hyperactivity and Impulsivity subscales (FBB-HKS, parents' ratings) (Table 5, see Figure 4). Other moderate correlations (with CPRS Global Score, BRIEF Behavioral Score) resulted in trends for this subgroup. In the group of poor performers, changes on the behavioural scales were unrelated to the magnitude of differentiation. Improvements according to teachers' ratings did not result in any significant correlation, even when subgroups were considered separately.

**Table 5:** Correlation coefficients between training performance (differentiation in transfer trials), improvement on behavioural scales, and parental support of the neurofeedback group

	**Differentiation Transfer**			**Parental support**
**Difference scores**	Total group (N = 17)	Good performers (N = 8)	Poor performers (N = 9)	Total group (N = 17)
**Parents' ratings**				
DSM IV (FBB- HKS)				
Hyperactivity	.11	.81 *	-.32	.34
Inattention	.10	.21	.45	.52 *
Impulsivity	.03	.75 *	-.35	-.02
				
BRIEF				
Behavioral Index	.23	.64	.32	.42
Metacognitive Index	.18	.31	.06	.35
CPRS				
Global Score	.17	.64	.12	.45
				
**Teachers' ratings**				
CTRS				
Global Score	-.16	.17	-.29	.54 *
BRIEF				
Behavioral Index	.07	.0	-.37	.25
Metacognitive Index	.18	.27	-.22	.43
				
**Neuropsychological tests**				
Alertness TAP *sd*	-.51 *	-.62	-.15	-.27
Go/nogo TAP *errors*	.17	.29	-.72 *	-.12
Score *correct*	-.33	.56	-.39	.21
Code transmission *correct*	-.13	.32	.13	-.05
D2 *concentration*	-.11	.14	.27	-.30
TMT *(time B-A)*	-.04	-.14	-.48	.32

* p < .05, BRIEF = Behavior Rating Inventory of Executive Function, CPRS = Conners' Parent Rating Sale, CTRS= Conners' Teacher Rating Scale, TAP = Test for Attentional Performance, D2 = D2 Test of Attention, TMT= Trail Making Test

Changes in neuropsychological test before and after the training were mostly unrelated to the ability to differentiate between negativity and positivity (Table 5). The only significant – but negative – correlation was found between the difference in the standard deviation of the Alertness task and the mean differentiation between positivity and negativity, which was more pronounced for the subgroup of good performers. This correlation can be explained by the fact that the children of the neurofeedback group did not improve on this task but rather showed a small but not significant increase of reaction time variability. Among the poor performers, improvement on the Go/nogo-task was inversely related to training performance. With exception of the TMT parameters, the remaining correlations, though not significant in this very small group, turned out to be positive for the good performers, which intuitively is in accordance with the expected direction of change associated with good achievement. One has to take into account, however, that difference scores may provide a biased result, as initially severely impaired participants have a better chance to improve.

### The importance of parents' support during neurofeedback training

In order to quantify the impact of the support provided by the parents during the neurofeedback training and during the intermediate five weeks without EEG-feedback training, parents contributions were categorized into "high" or "low" according to the involvement of the parents in the training and according to parents' self-evaluations during the interview. As a result, 9 children were categorized into a "high support" group and the remaining 8 into a "low support" group. Parents of the "low support group" gave different reasons why they had failed to support training efforts, ranging from "he (the child) would not accept" over "time constraints" to "there was no need to". Only one child of the low support group seemed to have made use of the transfer cards outside the therapeutic setting. In contrast, as indicated by parents, all the children of the high support group had systematically used transfer cards in daily life, especially when doing homework or during school exams. There was no evidence of a direct influence of high parental support on successful cortical control: Fifty percent of the good performers and 66 percent of the poor performers belonged to the "high-support group"(Table 4).

In a next step this categorical parental support variable was correlated with behavioural improvements. Difference scores of several scales showed moderate correlations with parental support, but only two of them were significant, the correlation with the Inattention score according to DSM IV (FBB-HKS) (parents' ratings) and the Conners' Global Index of the teachers' ratings (Table 5).

## Discussion

In the present study the effects of a SCP neurofeedback training were evaluated and compared to a group training program for children with ADHD. In contrast to previous studies, specificity of neurofeedback was assessed by multiple means, using a control group, computing correlations with EEG training success, and controlling for parental support as a mediating factor.

According to parents' ratings, both groups showed behavioural improvements over time, but the benefit of the neurofeedback training was clearly more pronounced, especially on scales of cognitive regulation (inattention and metacognitive abilities), whereas no advantage was found for the feedback training on behavioural regulation (e.g. inhibitory control, hyperactivity). According to teachers' ratings, some improvement was found after neurofeedback training, although minor compared to parents' estimate, whereas no improvement was observed after group therapy. These results are roughly in line with former studies on behavioural improvements after neurofeedback training (e.g[5,6,8,10]) or behavioural therapy (see [44]) in children with ADHD. While they confirm our hypothesis about the superiority of neurofeedback training effects on core symptoms, they leave open which factors mediate this difference.

On the neuropsychological tests both groups showed significant improvement on several tasks over time, but no advantage was observed for either group. Both groups improved on the inhibitory control task (Go/nogo), although group differences were significant here, with children of the neurofeedback group being more impaired than the group therapy children. Improvements on neuropsychological tests have been reported in several studies after neurofeedback training (e.g. [8,10]). As the group therapy did not aim at basics cognitive skills, one would have expected an advantage for neurofeedback training on neuropsychological improvement. On the basis of group evaluation alone it cannot be concluded to which extent cognitive improvements are due to rather unspecific effects of the treatment. Also, practice effects rather than cognitive improvement may have accounted here for the better test results in both groups at the second time of testing.

In a second step, the specific effects of neurofeedback training were evaluated separately. Children with ADHD learned to control cortical regulation and increased cortical activation (= negativation), whereas positivation did not improve significantly over time but was reliably present from the beginning. Similar findings have been reported by Leins et al. [8]. As the training principally aims at the controlled increase of negativity ("activation"), the major goal of the training was thus achieved. However, more than half of children failed to discriminate reliably between the generation of positivity and negativity without immediate feedback, suggesting that they did not learn to transfer the differentiation between the two cortical states into typical situations without neurofeedback. It has been reported in several studies that some participants fail to obtain satisfactory training results during EEG feedback or transfer (e.g. [5,10,19]), but the proportion of non-responders was generally much lower than in the present sample. Possibly, the SCP training, which is rather strategic in nature and prone to artefacts, does present more difficulties for children with ADHD than a neurofeedback training based on frequency ratios. Obviously, regulation was more easily achieved with direct feedback so that the transfer might have presented the main difficulty for many children at the end of the training program and not the regulation itself. Also, intraindividual variability was considerable and one should keep in mind that analyses based on mean amplitudes of activations are closer to performance estimations than to exact measurements. For the present sample we could not identify demographic, symptomatic or other characteristics which might have predicted poor transfer training performance. Considering the large number of non-responders in the group, it is not surprising that no significant correlation emerged when improvements of the total neurofeedback- group were correlated with the training performance (during EEG transfer). However, when the results of the good performers were entered separately into the analysis, sizeable correlations with indices of behavioural regulation (inhibition, impulsivity) appeared, and reached significance despite the small size of the subsample.

One may argue that the specific advantage of neurofeedback training compared to group therapy – improvements on scales of cognitive regulation according to parents and teachers – was not well explained by the specific effect of electrophysiological training. However, such a specific effect cannot be expected given that not even half of the neurofeedback participants managed to differentiate their cortical activation on transfer trials. An environmental variable, the amount of support provided by parents, accounted better for this general group effect. This measure was uncorrelated with the specific electrophysiological measures of training success. We assume that the training with transfer cards provided for parents and children an acceptable conceptualization on how to deal with problem situations and problem behaviour, regardless of the children's cortical state control. Also, parents were instructed to adopt principles of behavioural management and to give positive feedback in everyday situations. Together, this may be likened to an additional behavioural training by the parents, which contributed to increase self-attention and enhance self-management skills in attention-demanding situations. The importance of the parental style for training success has been described by Monastra et al. [4] for frequency training. One has to take into account that a training of SCPs involves far more than the control over electrophysiological processes and that the learning procedure itself may be more strategic by nature than in frequency training.

The results of the current study are the best explained by two additive effects: About half of the children, the good performers, learned to regulate cortical control during neurofeedback sessions and showed improved hyperactive/impulsive symptoms due to the specific effects of the training. In addition, a portion of the children continued to train their mental states at home with the help of their parents and improved in several cognitive-behavioural domains thanks to these non-specific effects of a behavioural training. There was no evidence for a systematic overlap of good performance and parental support in our small sample. The combination of these two effects – and possibly several others which we did not control for – may have led to the positive results of our study.

### Limitations of the study

The study presents certain limitations: the main limitations are the rather small number of participants and the incomplete randomization. Although differences between groups are statistically not significant – except for one test result -, treatment groups are not perfectly matched. This does not affect the general conclusions. However, results should be considered as preliminary. One may ask to which extent group therapy constitutes an appropriate control condition for neurofeedback training, given the many differences in the setting, method and design. In order to guarantee a better comparability, an individual behavioural therapy with concomitant parent education might constitute a viable alternative for future research, although important intervening factors, such as social feedback in the group setting, would be lost.

## Conclusion

Regarding our initial hypotheses, conclusions must be twofold: On one hand, behavioural improvements can be related to the effects of neurofeedback training and to specific electrophysiological mechanisms, at least in a subgroup of children who learned to regulate cortical activity. These effects seem to account especially for changes in behavioural regulation. On the other hand, the advantage found for neurofeedback compared to group therapy according to parents' and teachers' rating cannot be explained by electrophysiological mechanisms in the full group, but rather seems mediated by unspecific factors, such as parental support or certain properties of the therapeutic setting and content. These effects are mostly related to cognitive aspects of regulation.

## Competing interests

The author(s) declare that they have no competing interests.

## Authors' contributions

RD conceived the study, supervised the clinical training, was responsible for data collection and statistical analysis, and wrote the first draft of the paper. MS carried out most of the clinical neurofeedback training, was involved in the collection of the data, in their analysis and interpretation. MD contributed to data collection, analysis and interpretation, especially for the electrophysiological aspects. HH developed the training method, contributed to the design of the study and critically revised the final manuscript. DB contributed to the conception of the study, was responsible for the electrophysiological part of data collection and interpretation, and helped to draft the manuscript. HCS contributed to the conception of the study, continuously supervised the study and critically revised the final manuscript. All authors read and approved the final manuscript.
